# Magnetic properties of molecules on surfaces studied with scanning probe methods

**DOI:** 10.1093/nsr/nwag109

**Published:** 2026-02-23

**Authors:** Chao Li, Caimei Gong, Alexander Weismann, Fengqi Song, Yongfeng Wang, Richard Berndt

**Affiliations:** Institute of Atomic Manufacturing, Nanjing University, Nanjing 210089, China; Nanjing Institute of Atomic-Scale Manufacturing, Nanjing 211800, China; Nanjing Institute of Atomic-Scale Manufacturing, Nanjing 211800, China; Institut für Experimentelle und Angewandte Physik, Christian-Albrechts-Universität, Kiel 24098, Germany; Institute of Atomic Manufacturing, Nanjing University, Nanjing 210089, China; Nanjing Institute of Atomic-Scale Manufacturing, Nanjing 211800, China; Center for Carbon-Based Electronics and Key Laboratory for the Physics and Chemistry of Nanodevices, School of Electronics, Peking University, Beijing 100871, China; Institut für Experimentelle und Angewandte Physik, Christian-Albrechts-Universität, Kiel 24098, Germany

**Keywords:** molecular magnetism, scanning tunneling microscopy, spin–surface interaction, Yu–Shiba–Rusinov states

## Abstract

Magnetic molecules on surfaces provide atomically precise test beds for quantum magnetism and device concepts in spintronics and quantum information. Scanning probe techniques now resolve and control single-molecule spin states, their coupling to substrates and emergent many-body phenomena, including the Kondo effect and Yu–Shiba–Rusinov states. This review summarizes recent progress in combining molecular design, adsorption geometry and interfacial hybridization to create measurable spin anisotropy, excitations and correlations. We first recall the fundamentals of spin formation and surface-induced modifications, then survey key methodologies such as scanning tunneling microscopy, inelastic tunneling spectroscopy, pump–probe protocols, functionalized and superconducting tips, and magnetic exchange force microscopy. Case studies span single-ion magnets, metal-free Kondo-active molecules, molecular magnets on superconductors, and coupled spin architectures assembled by on-surface synthesis. Across these examples, we distill design rules for tuning exchange, anisotropy and charge transfer at the molecule–surface interface. We conclude with open challenges—reproducibility, room-temperature stability and multiscale theory—and outline opportunities in van der Waals integration, ultrafast spin dynamics and data-driven spectroscopy, charting a path from single-molecule experiments to programmable molecular spin devices.

## Teaser

Magnetic molecules on surfaces emerge as atomically precise testbeds for quantum magnetism, presenting opportunities to control Kondo resonances, Yu-Shiba-Rusinov states and engineered spin architectures via combined molecular design and scanning-probe methods.

## INTRODUCTION

Magnetic molecules provide a versatile platform for investigating fundamental quantum phenomena and for developing applications in molecular spintronics, quantum sensing and quantum information processing [1–3]. Prototype molecular systems have been proposed as qubits, memory units or logic elements that encode information in intrinsic spin states, magnetic anisotropy or exchange interactions at the single-molecule level [4–7]. Over the past four decades, molecular magnetism has evolved from a chemical curiosity into a broad interdisciplinary field bridging coordination chemistry, condensed matter physics and quantum information science [8–11].

**Figure 1.**
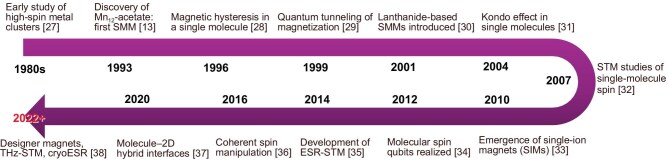
Key milestones in molecular magnetism. This timeline highlights pivotal discoveries and conceptual advances in the field of molecular magnetism, from early studies of high-spin metal clusters in the 1980s [27] to the realization of single-molecule magnets (SMMs) in the 1990s [13,28,29], and more recent progress in spin-resolved scanning probe techniques, coherent spin control and quantum molecular devices. Milestones include the discovery of Mn$_{12}$ acetate as the first SMM (1993) [13], the observation of quantum tunneling of magnetization (1996) [28], the development of lanthanide-based and single-ion magnets (2001–10) [30–33], and key advances in molecular spin qubits and electron spin resonance-scanning tunneling microscopy (ESR-STM; 2012–22) [34–38]. In recent years (post-2020), growing interest has focused on the integration of molecular magnets with *superconducting substrates*, enabling the study of Yu–Shiba–Rusinov states and topological excitations such as Majorana zero modes. In parallel, bottom-up *on-surface synthesis* strategies have enabled the construction of custom-designed magnetic molecules and networks with atomic precision, opening new directions in molecule-based quantum materials. Years indicate when a concept or effect became recognized in the field; references correspond to representative or widely cited publications.

The development of molecular magnetism can be traced to the 1980s, with the synthesis of high-nuclearity transition-metal clusters in which collective spin states and magnetic exchange interactions were first observed in discrete molecular units (Fig. 1) [12]. A major breakthrough came with the discovery of Mn$_{12}$ acetate as the first single-molecule magnet (SMM), which exhibited magnetic hysteresis of purely molecular origin [13]. Soon after, the observation of quantum tunneling of magnetization established the quantum-mechanical nature of these spin states [14]. The subsequent introduction of lanthanide-based SMMs and single-ion magnets (SIMs) greatly expanded the chemical design space for high-anisotropy molecules. In parallel, advances in experimental methodology—most notably scanning probe microscopy and electron paramagnetic resonance (EPR)—enabled the interrogation of individual spin centers with atomic precision.

More recently, new research frontiers have emerged. Magnetic molecules on superconducting substrates allow direct access to Yu–Shiba–Rusinov (YSR) states and engineered topological excitations [15]. In parallel, bottom-up on-surface synthesis strategies permit the creation of atomically precise nanographenes and radical frameworks with tailored spin architectures [16,17]. These developments position molecular magnetism not only as a platform for studying strongly correlated electron physics but also as a promising building block for future quantum technologies.

On surfaces, molecules often arrange into dense arrays and consequently interact strongly with their molecular environment and the substrate [18]. These interactions can stabilize or quench spin states, tune magnetic anisotropy through modified ligand fields [19] or give rise to many-body phenomena such as the Kondo effect [20] and YSR states [21]. Importantly, these surface-induced modifications are not merely perturbative but provide opportunities to engineer new electronic and magnetic states. Accessing and controlling such behavior requires experimental techniques with exceptional spatial, energetic and magnetic resolution [22].

Scanning probe methods have proven indispensable in this context. They not only visualize molecules with sub-ångström resolution but also enable spectroscopy of spin excitations, spin-polarized tunneling currents [22] and force-mediated magnetic exchange [23]. Hybrid approaches, such as superconducting tips, molecular functionalization or pump–probe protocols, have further expanded their reach [24,25], enabling the direct observation of spin textures, spin transitions and ultrafast relaxation processes [26]. While several excellent reviews address molecular magnetism more broadly or focus on transport through single-molecule junctions, this review concentrates on the insights into magnetic molecules on surfaces enabled by scanning-probe methods, such as scanning tunneling microscopy (STM) and atomic force microscopy (AFM). We do not attempt to cover transport in break-junction devices, bulk magnetic properties of molecular crystals or predominantly theoretical studies. Instead, we present recent experimental advances that directly link atomic-scale probe measurements to the understanding, design and prospects of molecular-spin devices at surfaces.

In this review, we provide an overview of how scanning probe methods have deepened our understanding of molecular magnetism on surfaces. We first summarize the fundamental mechanisms governing spin states in low-dimensional environments [2,9], focusing on hybridization, symmetry breaking and charge transfer at molecule–surface interfaces [39]. We then describe the operating principles and applications of scanning probe techniques—ranging from spin-polarized STM and inelastic tunneling spectroscopy to emerging approaches such as superconducting-tip Andreev spectroscopy. The core sections present case studies illustrating how molecular design and surface environments jointly determine spin-related phenomena: single-ion magnets with tunable anisotropy [19], open-shell molecules exhibiting gate-controlled Kondo resonances [40] and YSR states induced by magnetic molecules on superconductors [21]. We also highlight the construction of coupled spin systems—dimers, chains and networks [41–43]—and the emergence of complex magnetic textures in nanographenes and radical frameworks prepared by on-surface synthesis [44,45].

Despite rapid progress, several challenges remain. Experimental studies are typically restricted to cryogenic ultra-high vacuum conditions [46], while theoretical modeling must capture strong correlation, substrate screening and dynamical effects [47]. Furthermore, reliable integration of magnetic molecules into functional devices requires strategies that balance stability, tunability and scalability. We conclude by outlining future directions: the integration of molecular magnets with two-dimensional (2D) materials and van der Waals heterostructures [48], the development of ultrafast scanning-probe techniques for time-resolved spin dynamics and the deployment of machine learning to classify spin states and spectral features [49]. As scanning probe microscopy continues to evolve, it is poised to play a central role in unraveling and harnessing quantum magnetism at the single-molecule level.

## FUNDAMENTALS OF MOLECULAR MAGNETISM ON SURFACES

Molecular magnetism arises from the spin and orbital configurations of unpaired electrons within a molecule. Representative systems include organic radicals ($S=1/2$), transition-metal complexes with higher spin states ($S=1, 3/2, 2$) and single-molecule magnets. While the inherent magnetic properties (e.g. the spin ground state) are defined by the molecular structure itself, the manifestation of key phenomena such as zero-field splitting, slow magnetic relaxation and magnetic hysteresis requires a fixed molecular orientation to break rotational symmetry. This orientation is achieved in crystalline lattices, where these anisotropies are stabilized and tuned by well-defined molecular symmetry and ligand fields. In crystals, spin-orbit coupling and the crystal field lift the degeneracy between spin orientations, thereby establishing the magnetic anisotropy. Adsorption on solid surfaces modifies and often enhances the resulting anisotropy through ligand-field distortion, charge transfer and coupling to conduction electrons or phonons [50,51]. Understanding such perturbations is central to realizing molecular spintronic and quantum information devices.

### Magnetic anisotropy and spin Hamiltonian

At the single-molecule level, magnetic anisotropy governs the stability of spin states. A widely used effective Hamiltonian to describe this anisotropy is given by


**Figure 2.**
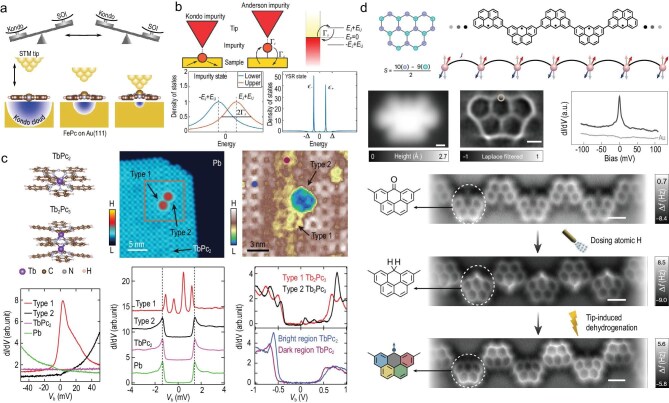
Correlated magnetic states of surface-supported molecules. (a) Tip-controlled hybridization and anisotropy. Vertical displacement of the Fe$^{2+}$ ion in FePc (by approaching the STM tip) distorts the ligand field, increases the Fe–Au distance and reduces $3d$–substrate hybridization $\Gamma$. This tunes the exchange *J* in the Kondo Hamiltonian and effectively modifies the anisotropy parameters in $H_{\mathrm{ani}}$ [Equation (1)], driving a crossover between Kondo and spin-excitation spectra. Adapted with permission from Hiraoka *et al.* [54]. (b) YSR formation principle. Schematic of an impurity level in the normal state compared with a superconducting host: in the latter, exchange with the condensate creates in-gap YSR states at energies set by Equation (2). Adapted with permission from Huang *et al.* [58]. (c) Orbital-resolved YSR channels. STM topograph and $\mathrm{d}I/\mathrm{d}V$ maps for TbPc$_2$/Tb$_2$Pc$_3$ on Pb(111) reveal multiple, spatially distinct YSR resonances. Tip-sensitive and robust peaks indicate ligand-spin and charge-fluctuation channels, respectively. Adapted with permission from Xia *et al.* [57]. (d) Heisenberg spin chains from radicals. STM and non-contact atomic force microscopy (nc-AFM) images, together with $\mathrm{d}I/\mathrm{d}V$ spectroscopy, establish $S=1/2$ chains whose finite-size gaps follow the antiferromagnetic Heisenberg model [Equation (3)] and scale approximately as $1/L$. Scale bars: 0.5 nm. Adapted with permission from Zhao *et al.* [59].

(1) \begin{eqnarray*}
H_{\mathrm{ani}} = D S_z^2 + E (S_x^2 - S_y^2),
\end{eqnarray*}

where *D* and *E* parameterize axial and transverse anisotropy, respectively, and $S_x$, $S_y$, $S_z$ are the spin operators. The sign of *D* determines whether orienting the magnetic moment is easy along an axis (easy-axis regime, $D<0$) or within a plane (easy-plane regime, $D>0$). The parameter *E*, on the other hand, mixes spin sublevels and influences the quantum tunneling of magnetization [52]. In a magnetic field, an additional Zeeman term modifies the level scheme. Spin excitations, i.e. transitions between the eigenstates of this Hamiltonian lead to inelastic tunneling processes that can be probed by tunneling spectroscopy. On surfaces, both *D* and *E* are affected by hybridization with the substrate and by the adsorption geometry. For adatoms, the magnetic anisotropy is often substantially enhanced. Tip-induced distortions enable control over these parameters (see Fig. 2a, where vertical Fe displacement modulates the ligand field and hybridization).

### Kondo effect and exchange coupling

When a molecular magnetic moment is exchange-coupled to itinerant electrons of the substrate, a new ground state may arise. For antiferromagnetic coupling, the system forms a many-body singlet state with total spin $S=0$ below the Kondo temperature $T_\mathrm{K}$, i.e. the molecular moment becomes screened by the conduction electrons. This gives rise to a narrow resonance at the Fermi level, which can be detected by STM as a zero-bias feature in the differential conductance $\mathrm{d}I/\mathrm{d}V$ [53]. The width of the resonance is proportional to the Kondo temperature $T_\mathrm{K}$. In complex cases like Fe phthalocyanine (FePc; throughout this review, ‘Pc’ abbreviates ’phthalocyanine’) on Au(111), two Kondo temperatures ($T_\mathrm{K}\sim 150$ K and $T_\mathrm{K}\sim 3$–4 K) may be required to model the Kondo effects mediated by different orbitals or by hybridization with the STM tip [54]. Tip approach (or vertical displacement of the spin-carrying ion) predominantly reduces the hybridization $\Gamma$ between the Fe $3d$ orbitals and the Au(111) substrate, thereby effectively tuning *J*. This reduction in substrate hybridization drives a crossover from Kondo-dominated spectra to spin-excitation features—precisely the control route sketched in Fig. 2a.

### Yu–Shiba–Rusinov states

On a superconducting surface, the localized spin of magnetic molecules perturbs the Cooper-pair condensate and may give rise to YSR states inside the superconducting energy gap. These states manifest as symmetric subgap resonances in $\mathrm{d}I/\mathrm{d}V$ spectra, with energies that are highly sensitive to the spin magnitude *S* and to the molecule-substrate hybridization $\Gamma$, which governs the exchange coupling *J*. The YSR energies can be approximated by Equation (2) below, providing a simple relation between the coupling strength and the subgap state energy. A quantum phase transition can occur when the exchange coupling exceeds a critical threshold, driving the system from a doublet ground state to a screened singlet. This competition between Kondo-like screening and superconducting pairing exemplifies the rich many-body physics of magnetic–superconducting interfaces [55], now routinely resolved by low-temperature STM with superconducting tips (see Fig. 7 below). The energies of YSR states can be approximated by


(2) \begin{eqnarray*}
E_{\mathrm{YSR}} = \Delta \frac{1-\alpha ^2}{1+\alpha ^2}, \qquad \alpha = \pi \rho _0 J S,
\end{eqnarray*}

where $\Delta$ is the gap parameter, $\rho _0$ is the normal-conducting density of states at the Fermi level, *J* is the exchange coupling strength and *S* is the spin quantum number of the magnetic impurity. The dimensionless coupling strength $\alpha$ is controlled by the normal-state density of states $\rho _0$ and the exchange coupling *J* of the localized spin *S* to the substrate electrons [56]. In conductance spectra acquired with normal-conducting (superconducting) tips, the resonances may be observed at voltages $\pm E_{\mathrm{YSR}}/e$ ($\pm (E_{\mathrm{YSR}}+\Delta _\mathrm{T})/e$), where $\Delta _\mathrm{T}$ is the gap parameter of the tip. In complex molecules, multiple frontier orbitals can host distinct YSR channels. For example, while TbPc$_2$ on Pb(111) shows featureless low-bias spectra indicating a quenched ligand spin, type-1 Tb$_2$Pc$_3$ molecules exhibit two pairs of orbital-resolved YSR resonances that respond differently to STM tip proximity. These features are assigned to a ligand-localized spin channel arising from a singly occupied molecular orbital and to a valence-fluctuating (‘charge-fluctuation’) orbital whose occupancy is not fixed owing to charge transfer and hybridization with the substrate (Fig. 2b and c) [57,58].

**Figure 3.**
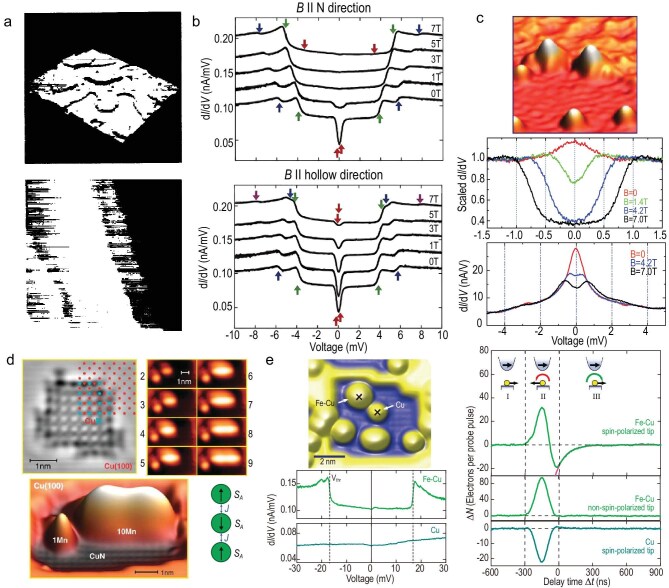
STM-based techniques for probing atomic and molecular spins. (a) Spin-polarized STM: real-space magnetic contrast on Cr(001), showing antiferromagnetic rows with step-height modulation. Adapted with permission from Wiesendanger *et al.* [60]. (b) STM-IETS of an individual magnetic atom: field-dependent $\mathrm{d}I/\mathrm{d}V$ spectra on a single Fe atom, showing spin-excitation steps and magnetic anisotropy. Adapted with permission from Hirjibehedin *et al.* [32]. (c) Kondo resonance spectroscopy: Mn atoms near oxide boundaries show Fano-shaped zero-bias peaks reflecting spin screening. Adapted with permission from Heinrich *et al.* [61]. (d) Atomic manipulation and engineered chains: atom-by-atom assembly of Mn chains on CuN/Cu(100) reveals antiferromagnetic coupling consistent with the Heisenberg model. Adapted with permission from Hirjibehedin *et al.* [62]. (e) Pump–probe STM: ultrafast voltage pulses applied to an Fe–Cu dimer measure spin relaxation ($T_1 \sim 87$ ns) at $B=7$ T, revealing decoherence pathways. Adapted with permission from Loth *et al.* [63].

**Figure 4.**
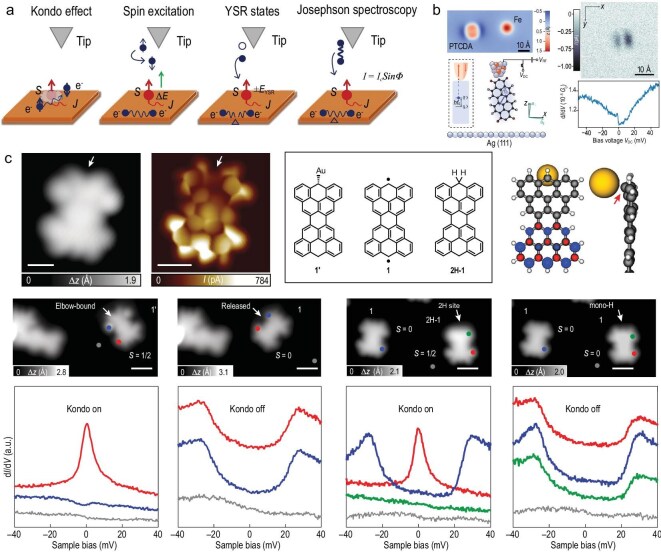
Hybrid scanning probe techniques for molecular magnetism. (a) Superconducting probe phenomena: Kondo resonances, spin excitations, Yu–Shiba–Rusinov states and Josephson/Andreev processes illustrate spin–superconductor interactions. (b) Molecular quantum sensor: PTCDA attached to an Fe tip acts as a spin qubit for ESR-based local-field sensing. Adapted with permission from Esat *et al.* [73]. (c) On-surface synthesis: frustrated nanographenes (Clar’s goblet, C$_{38}$H$_{16}$) stabilize emergent magnetic edge states. Scale bars: 0.5 nm (the first row); 1 nm (the second row). Adapted with permission from Mishra *et al.* [45].

**Figure 5.**
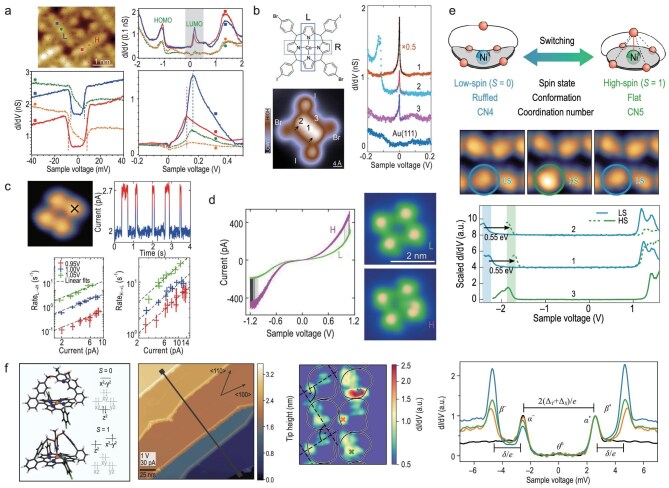
Control and switching of single-molecule spins on surfaces. (a) Fe(II) 5,10,15,20-tetrakis(4′-bromophenyl)porphyrin (FeTBrPP) molecules self-assemble on Au(111) in L and H orientations with saddle-shaped geometry. STM and $\mathrm{d}I/\mathrm{d}V$ spectra reveal spin-flip excitations and site-specific electronic structures. HOMO: highest occupied molecular orbital; LUMO: lowest unoccupied molecular orbital. Adapted with permission from Meng *et al.* [74]. (b) Isolated CoTPPBr$_2$I$_2$ molecules exhibit two chiral states, L and R, defined by upward or downward pyrrole bending. STM and low-bias $\mathrm{d}I/\mathrm{d}V$ spectra show site-specific electronic features. Adapted with permission from Meng *et al.* [75]. (c) Tetramer switching dynamics. STM time series show rapid current jumps between low and high states. Switching rates are proportional to the current, demonstrating reversible single-molecule switching. Adapted with permission from Johannsen *et al.* [76]. (d) Voltage sweeps induce abrupt L$\leftrightarrow$H transitions in a tetramer, producing hysteresis. STM images show pristine and switched molecules, with the H state displaying intramolecular contrast. Adapted with permission from Johannsen *et al.* [77]. (e) Interlocked complexes on Ag(111) reversibly switch between low-spin (CN$_4$) and high-spin (CN$_5$) states via pyridine coordination. STM topographs and $\mathrm{d}I/\mathrm{d}V$ spectra reveal height changes and HOMO shifts. Adapted with permission from Köbke *et al.* [24]. (f) Hairclip-porphyrin molecules (5,15-(2′-(4″-(4″′-methylpyridin-3$^{\prime \prime }$,$5^{\prime \prime }$-ylen)-oxymethylphenyl) phenyl)-10,20-bis(2,3,4,5,6-pentafluorophenyl)-Ni(II)-porphyrin) on Pb(100) occupy step edges. Low-bias STM images and $\mathrm{d}I/\mathrm{d}V$ spectra show dumbbell patterns and spin/coherence peaks, with the A$^{\ast }$ state exhibiting higher conductance. Adapted with permission from Treichel *et al.* [78].

**Figure 6.**
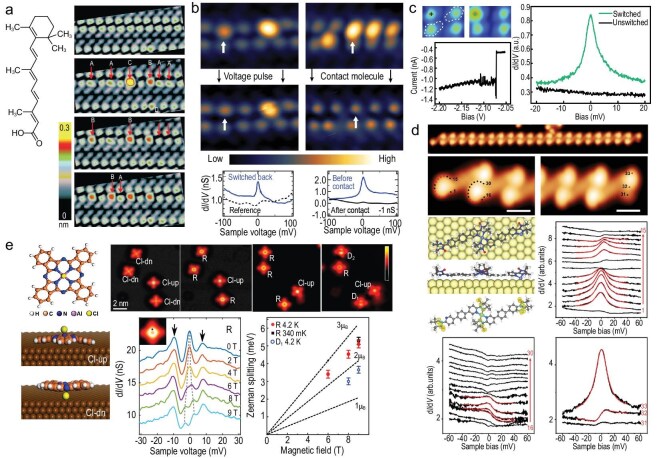
Kondo-active molecules (without a magnetic ion center). (a) Striped dimers of retinoic acid in their pristine state; selected molecules are switched into distinct states by voltage pulses. Adapted with permission from Karan *et al.* [21]. (b) STM images show pristine and switched molecules; $\mathrm{d}I/\mathrm{d}V$ spectra reveal electronic changes and the effects of tip contact. Adapted with permission from Bocquet *et al.* [44]. (c) Voltage pulses switch target molecules within hydrogen-bonded dimers; current jumps indicate dehydrogenation, as confirmed by $\mathrm{d}I/\mathrm{d}V$ spectra. Adapted with permission from Zhong *et al.* [79]. (d) Complete chains and chain ends are imaged; DFT spin densities show localization, and $\mathrm{d}I/\mathrm{d}V$ spectra fitted with Fano functions reveal local electronic and spin features. Scale bars: 0.5 nm. Adapted with permission from Wang *et al.* [20]. (e) ClAlPc on Cu(100) switches from a Cl-down configuration to rotated states upon application of voltage pulses; $\mathrm{d}I/\mathrm{d}V$ spectra reveal satellite peaks and magnetic-field-dependent zero-bias splittings consistent with 1–3 $\mu _B$. Adapted with permission from Li *et al.* [80].

**Figure 7.**
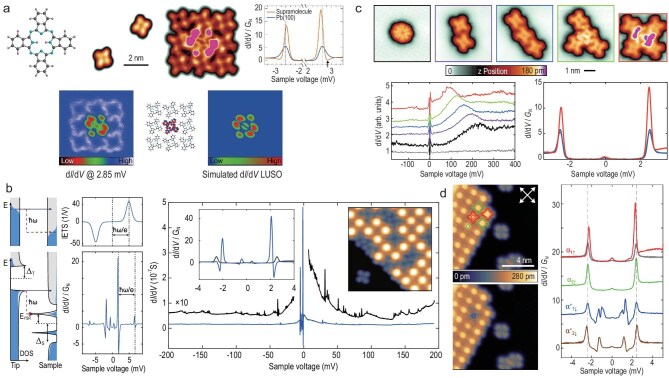
Yu–Shiba–Rusinov molecules on superconducting substrates. (a) STM images and $\mathrm{d}I/\mathrm{d}V$ spectra of H$_2$Pc on Pb(100) show molecular states, YSR resonances and orbital patterns consistent with density functional theory (DFT). Adapted with permission from Homberg *et al.* [81]. (b) Tunneling schematics and spectra illustrating YSR states and vibrational excitations, together with experimental $\mathrm{d}I/\mathrm{d}V$ spectra of PbPc on Pb(100) showing YSR peaks ($\pm$2 mV) and vibrational features. LUSO: lowest unoccupied supramolecular orbital. Adapted with permission from Homberg *et al.* [82]. (c) STM images of AlPc cluster formation; corresponding $\mathrm{d}I/\mathrm{d}V$ spectra show LUMO shifts with added neighbors, and YSR states emerge for the pentamer. Adapted with permission from Li *et al.* [83]. (d) STM image of a SnPc$\uparrow$ island showing $\alpha _1$/$\alpha _2$ molecules, with $\alpha _1$ switched to $\alpha _1^{*} \downarrow$; $\mathrm{d}I/\mathrm{d}V$ spectra compare their spin states to the substrate. Adapted with permission from Banerjee *et al.* [84].

### Intermolecular exchange and spin chains

Coupled molecules realize model Hamiltonians for low-dimensional magnetism. For chains of $S=1/2$ radicals, the magnetic interactions can be captured by a simple Heisenberg Hamiltonian,


(3) \begin{eqnarray*}
H_{\mathrm{Heis}} = J \sum _{i} \mathbf {S}_i \cdot \mathbf {S}_{i+1},
\end{eqnarray*}

where *J* is the exchange coupling constant and $\mathbf {S}_i$ is the spin operator at site *i*. This Hamiltonian includes only nearest-neighbor interactions, with antiferromagnetic ($J>0$) or ferromagnetic ($J<0$) coupling depending on direct orbital overlap and substrate-mediated interactions. Spectroscopy reveals finite-size spin gaps scaling approximately as $1/L$ for chain length *L*, converging toward quasi-continuous excitation bands for longer chains [59]. On-surface-synthesized radical chains provide clean realizations of this physics with atomically resolved structure and spectra (see Fig. 2d).

### Role of the adsorption geometry

The geometry of a complex and its anchoring to the substrate determine its magnetic properties. Different adsorption sites alter ligand-field splittings; anchoring groups affect the hybridization strength; a molecular tilt modifies the overlap of a molecular $\pi$-electron system with substrate states. Consequently, the same molecular species can display a Kondo resonance in one geometry and spin excitations in another, underscoring the high sensitivity of spin phenomena to the atomic-scale configuration (compare the tuning routes illustrated in Fig. 2a–c).

## SCANNING PROBE TECHNIQUES FOR MAGNETIC CHARACTERIZATION

### STM-based methods

Over the past decades, the capabilities of STM have been extended to resolve magnetic order, spin excitations, correlated many-body states and ultrafast dynamics. Figure 3 summarizes some case studies.

#### Spin-polarized STM

Spin-polarized STM was comprehensively reviewed by Bode [64] and Wiesendanger [65]. The method relies on the fact that the tunneling current between two ferromagnetic electrodes depends on their respective spin polarizations. The current therefore contains information about the magnetization of the sample, which can be fully exploited if the tip’s spin polarization and orientation are known. However, the method requires magnetic electrodes; although at low temperatures and in a sufficiently large magnetic field, even a single magnetic atom at the tip apex is sufficient [64]. The technique has not been widely applied to magnetic molecules, presumably because ferromagnetic substrates tend to interact strongly with adsorbates [66].

#### Shot-noise spectroscopy with STM

Shot-noise STM extends conventional tunneling spectroscopy by measuring the intrinsic current fluctuations that arise from the discrete nature of electron transport. Unlike standard STM, which probes the time-averaged tunneling current, shot-noise STM is sensitive to electron correlations and quasiparticle statistics at the atomic scale. This technique has been successfully employed to distinguish between different charge configurations, to probe spin-dependent scattering processes and to reveal many-body effects that are invisible in the time-averaged current. By combining submolecular spatial resolution with access to electronic noise spectra, shot-noise STM provides a powerful and complementary tool for characterizing the quantum magnetism of individual molecules and nanostructures on surfaces [67–69].

#### Inelastic electron tunneling spectroscopy

Inelastic electron tunneling spectroscopy (IETS) probes spin excitations when tunneling electrons provide the energy required for transitions between spin sublevels, which may be described using the effective Hamiltonian [Equation (1)]. Experimentally, spin excitations manifest as step-like increases in $\mathrm{d}I/\mathrm{d}V$ (and corresponding peaks in $\mathrm{d}^2I/\mathrm{d}V^2$). STM–IETS of Fe and Mn atoms shows such spectral fingerprints at bias voltages corresponding to the spin-excitation energies, and by analyzing their field dependence and intensities, anisotropy parameters and selection rules were determined (Fig. 3b) [32].

#### Kondo resonance spectroscopy

In scanning tunneling spectroscopy (STS), the Kondo effect appears as a resonance near the Fermi level, with its width related to the Kondo temperature $T_\mathrm{K}$. This resonance can be quantitatively described by a Fano line shape


(4) \begin{eqnarray*}
\frac{\mathrm{d}I}{\mathrm{d}V}(E) \propto \frac{(q+\epsilon )^2}{1+\epsilon ^2}, \qquad \epsilon = \frac{E - E_0}{\Gamma },
\end{eqnarray*}

where ${\mathrm{d}I}(E)/{\mathrm{d}V}$ is the differential conductance as a function of the energy *E* relative to the Fermi level, *q* is the Fano asymmetry parameter, $E_0$ is the resonance energy and $\Gamma$ is the resonance half-width. For example, Heinrich *et al.* [61] reported pronounced Kondo resonances for Mn adatoms on insulating Cu$_2$N layers and near oxide boundaries, which they fitted with Fano functions to determine $T_\mathrm{K}$ (Fig. 3c). While the Fano line shape originates from a Lorentzian, Frota showed that the Kondo resonances in renormalization group (NRG) spectra in fact follow an inverse square-root behavior, which can be expressed as


(5) \begin{eqnarray*}
\rho (E) \propto \Re \bigg [ \sqrt{\frac{i\Gamma }{E - E_0 + i\Gamma }} \bigg ].
\end{eqnarray*}

Here, $\rho (E)$ represents the local density of states (proportional to $\mathrm{d}I/\mathrm{d}V$). This expression also provides better agreement with Kondo resonances measured using STS [70]. Later, a generalized line-shape function was derived that, depending on the chosen parameter, reproduces both Fano and asymmetric Frota line shapes [71]. For experimental data analysis, a practical guideline emerges: the choice between Fano and Frota fits depends on the experimental resolution and temperature. Specifically, Fano fits are adequate when thermal or instrumental broadening masks the intrinsic Kondo line shape, while Frota fits are preferred in high-resolution, very-low-temperature spectra where the many-body asymmetric tail is resolvable.

#### Atomic manipulation and engineered spin chains

Beyond spectroscopy, STM manipulation allows the controlled construction of model spin systems. Individual atoms can be picked up, repositioned and deposited onto specific lattice sites with atomic precision, enabling the engineering of custom spin lattices governed by the Heisenberg Hamiltonian [Equation (3)]. A landmark example is the assembly of Mn chains on Cu$_2$N/Cu(100), which exhibited antiferromagnetic exchange and finite-size excitation gaps consistent with one-dimensional quantum magnetism (Fig. 3d) [62]. This bottom-up approach transforms STM into a laboratory for quantum simulations.

#### Pump–probe STM

Time resolution was introduced with pump–probe STM, in which a pair of voltage pulses with delay $\tau$ excites and probes the spin state. The transient current $I(\tau )$ in pump–probe STM experiments decays exponentially,


(6) \begin{eqnarray*}
I(\tau ) \propto \exp (-\tau /T_1),
\end{eqnarray*}

where $T_1$ is the spin relaxation time. Loth *et al.* [63] measured $T_1 \approx 87$ ns for an Fe–Cu dimer at $B=7$ T (Fig. 3e). Such pump–probe measurements are important because they reveal energy-relaxation channels at the atomic scale and set an upper bound on coherence lifetimes. However, the operational figure of merit for qubit gates is the phase-coherence time $T_2$, which is related to $T_1$ as


(7) \begin{eqnarray*}
\frac{1}{T_2}=\frac{1}{2T_1}+\frac{1}{T_\phi },
\end{eqnarray*}

where $T_\phi$ denotes pure dephasing. Because pure dephasing can dominate, $T_2$ is often shorter than $T_1$. Therefore, complementary phase-sensitive measurements are required to directly assess qubit-grade coherence on surfaces. The STM-based techniques represent a unique toolbox for molecular magnetism at surfaces. At the same time, these advanced techniques require high sample quality, which is typically achieved only in ultra-clean environments.

### Emerging complementary techniques

A new generation of *complementary scanning probe techniques* has emerged, integrating STM principles with modalities such as force detection, molecular functionalization, superconducting probes and on-surface synthesis. These approaches extend spatial, spectral and functional reach beyond conventional tunneling spectroscopy, enabling access to spin phenomena on insulating surfaces, exploration of many-body correlations and programmable assembly of exotic magnetic architectures (Fig. 4). Force-based approaches such as magnetic exchange force microscopy have demonstrated atomic-resolution magnetic contrast on insulating substrates [72]. Magnetic exchange force microscope (MExFM) enables the detection of short-range magnetic exchange forces and vectorial spin mapping on insulating materials—a capability beyond the reach of current-based spectroscopies.

#### Molecular quantum sensors on STM tips

By attaching a PTCDA (perylene-3,4,9,10-tetracarboxylic dianhydride) molecule to an Fe-modified tip, Esat *et al.* [73] engineered a two-level system that could be coherently addressed by electron spin resonance (ESR) and read out via magnetoresistance (Fig. 4b). This hybrid probe enabled mapping of local magnetic and electric fields with sub-ångström spatial resolution and neV-scale energy precision. Such molecular quantum sensors combine atomic-scale spatial resolution with coherent ESR control and neV energy sensitivity, enabling quantum operations and measurements of single-spin coherence that are inaccessible to conventional, incoherent tunneling spectroscopy.

#### On-surface synthesis of magnetic nanostructures

On-surface synthesis of magnetic nanostructures provides a complementary route by directly constructing spin networks with atomic precision. Mishra *et al.* [45] synthesized topologically frustrated nanographenes that stabilized unconventional edge-localized spin states (Fig. 4c). This approach transcends the passive observation of pre-adsorbed molecules. It allows for the bottom-up fabrication of spin architectures with designed exchange couplings and symmetries, thereby creating and probing correlated quantum phases and frustration effects that cannot be realized by studying spontaneously adsorbed species alone.

These hybrid techniques establish a versatile toolbox for probing and controlling spin physics at the single-molecule level.

## CASE STUDIES OF MOLECULAR MAGNETIC PROPERTIES ON SURFACES

### Single-ion magnets (e.g. transition-metal phthalocyanines/porphyrins)

SIMs tethered to surfaces merge the quantum nature of transition-metal spin centers with the chemical tunability of phthalocyanine or porphyrin ligands. These $\pi$-conjugated macrocycles impose strong ligand fields, giving rise to zero-field splitting and sizable magnetic anisotropy—key ingredients for stabilizing spin states and enabling information encoding at the molecular scale. Upon adsorption to metallic substrates, however, symmetry breaking, charge redistribution and orbital hybridization substantially alter these properties, modulating spin relaxation pathways and bistability.

#### Geometry-controlled anisotropy

FeTBrPP molecules on Au(111) (Fig. 5a) exemplify how the adsorption geometry tunes magnetic parameters. IETS revealed that different conformations shift the axial anisotropy $|D|$ from ${\sim }6.3$ meV to ${\sim }8.4$ meV, while preserving the $S=1$ ground state of the isolated molecule. According to DFT results, the anisotropy change may be attributed to a redistribution of electron density between the $d_{xz}/d_{yz}$ and $d_{z^2}$ orbitals, highlighting a route to control the magnetic anisotropy without altering the total spin [74].

#### Screening and Kondo effect

On the same surface, $\mathrm{CoTPPBr_{2}I_{2}}$ (cobalt(II)-5,15-bis(4’-bromophenyl)-10,20-bis(4’-iodophenyl)porphyrin) molecules show a strong sensitivity of their Kondo temperature $T_\mathrm{K}$ to the adsorption position and orientation on the Au(111) substrate (Fig. 5b), with $T_\mathrm{K}$ varying from ${\sim }8$ to 250 K. DFT calculations indicate that this drastic variation is consistent with subtle differences in molecule–substrate hybridization [75].

#### Intermolecular steric interactions

Electron injection into spin-crossover complexes can induce reversible switching between spin states. $\mathrm{Fe(H_2B(pyrazole){-}(pyridylpyrazole))_2}$ on Ag(111) is an intriguing example, as the complexes self-assemble into tetramers. Remarkably, two out of the four molecules can be toggled between $S=0$ and $S=2$ states (Fig. 5c) [76]. The absence of switching in the two molecules has been attributed to steric hindrance: the pyrazole groups of the immutable molecules are blocked by adjacent pyridine units, preventing the structural change concomitant with spin crossover. The spin-state switching exhibits hysteresis (Fig. 5d), demonstrating non-volatile bistability controllable at the single-molecule level [77].

#### Coordination-induced spin switching

A different strategy for implementing feedback between the molecular geometry and the spin state involves the mechanical motion of an axial ligand strapped to the macrocycle of a Ni porphyrin. The coordination of the ligand, and the concomitant planarity of the macrocycle, determine the Ni spin state. By partially decoupling the switching motif from the substrate, this design preserves bistability even on metal surfaces (Fig. 5e) [24]. Switching is induced by short voltage or current pulses from the STM tip. On a superconducting Pb(100) substrate, chains of this Ni-porphyrin derivative were shown to carry $S=1$ with tilted anisotropy axes. Spin-flip spectroscopy identified magnetic excitations, while bias-induced changes in appearance did not correspond to a true spin transition—unlike the behaviour of the same molecules on Ag(111) (Fig. 5f) [78]. This contrast highlights the strong influence of the substrate on the spin-crossover dynamics.

Adsorption geometry, spin crossover and intramolecular coordination thus emerge as versatile control parameters for stabilizing, tuning and switching spin states in surface-anchored SIMs. They showcase the versatility of molecular spins as functional building blocks for nanoscale devices.

### Kondo-active organic molecules without magnetic ions

While the Kondo effect is conventionally associated with transition-metal or rare-earth ions carrying localized moments, recent experiments have demonstrated that *metal-free organic molecules* on metals can also exhibit Kondo resonances.

#### Charge-transfer-induced radicals

Interfacial charge transfer is a direct route for converting closed-shell molecules into radicals. For example, the stable organic radical 1,3,5-triphenyl-6-oxoverdazyl shows a zero-bias Kondo resonance on Au(111) [87], while in other systems similar resonances arise from reaction-induced bond rearrangements or from orbital degeneracies within conjugated $\pi$ frameworks (Fig. 6).

#### Reactivity-driven spin formation

Hydrogen abstraction can also create localized moments. Karan *et al.* [21] showed that retinoic acid (ReA) on Au(111) can be manipulated to acquire an unpaired spin, signaled by a zero-bias resonance in conductance spectra (Fig. 6a). However, DFT modeling was unable to reproduce a localized spin in the adsorbed molecule. Importantly, high-field tunneling spectroscopy in the same work revealed Zeeman splitting of the zero-bias resonance, providing conclusive evidence for its magnetic origin. Subsequent modelling by Bocquet *et al.* [44] established that a sigmatropic hydrogen shift facilitates dehydrogenation, converting the closed-shell compound into a radical state (Fig. 6b).

In a similar vein, Zhong *et al.* [79] prepared close-packed arrays of dimers of (2E,4E)-3-methyl-5-(2,6,6-trimethylcyclohex-1-enyl) penta-2,4-dienoic acid (DIA) on Au(111). Negative voltage pulses (${\approx }-2.1$ V) applied via the STM tip reversibly toggle molecules between non-magnetic and Kondo-active states, as evidenced by abrupt current jumps in the tunneling current (Fig. 6c). This work establishes controlled chemical reactivity as an effective means for tuning molecular magnetism and demonstrates its potential for ultrahigh-density, single-molecule information storage at cryogenic temperatures.

#### Coordination-dependent spin quenching

The bonding configuration can determine whether a molecule supports a stable radical state with an associated $S=1/2$ magnetic moment. Wang *et al.* [20] studied nitroxide radicals on Au(111) and found that the coordination to metal centers either quenches or preserves the $S=1/2$ spin, depending on whether the nitroxide oxygen participates in bonding (Fig. 6d). DFT spin densities and Fano fits of $\mathrm{d}I/\mathrm{d}V$ spectra confirm the localization of spins at chain ends.

#### Orbital Kondo resonances

Beyond spin degrees of freedom, orbital degeneracies can also give rise to Kondo-like features. While such degeneracies are prone to being lifted by molecular distortions—e.g. due to a static Jahn-Teller effect—an *orbital Kondo effect* has been identified for ClAlPc on Cu(100). It manifests as a zero-bias resonance that exhibits an unusually large Zeeman splitting in magnetic fields, along with satellite peaks showing characteristics of a dynamical Jahn-Teller effect (Fig. 6e) [80].

Inelastic orbital excitations arising from a static Jahn-Teller effect have previously been proposed for satellites observed from Mn-phthalocyanine [88]. An orbital Kondo effect has also been invoked for an iron(II) atom [89,90]. Spin phenomena in purely organic systems emerge through diverse mechanisms—including charge transfer, bond rearrangement, electrical perturbations, coordination effects and orbital degeneracy—extending molecular magnetism beyond traditional metal-ion complexes and broadening the design space for molecular-scale spin devices.

### Magnetic molecules on superconducting substrates

#### Making molecules magnetic via electrostatic interaction

As first reported for phthalocyanine (H$_2$Pc) on Pb(100) (Fig. 7a), interactions between neighboring molecules can induce electrostatic shifts of the LUMO, leading to partial occupation and thereby generating a molecular spin. This effect naturally occurs in self-assembled islands but can also be deliberately tuned in artificially constructed arrays. It has also been reported for on-surface synthesized Al-phthalocyanine on Pb(100) (Fig. 7c) [83] and for PbPc [91]. In the H$_2$Pc case, tautomerization of pyrrolic hydrogen atoms can be induced in selected molecules to invert their electrostatic quadrupole field, thereby shifting YSR resonances of individual molecules [81].

#### A spin switch based on the image potential

In the examples above, the LUMO energy was downshifted by positioning molecules with peripheral hydrogen atoms exhibiting a partial positive charge in close proximity. For non-planar, shuttlecock-shaped molecules such as PbPc and SnPc, two states with the central atom located either below or above the macrocycle exist. For SnPc, transitions between these conformers can be induced in individual molecules and concomitantly change the spin state of the complex, as evidenced by spectroscopy of YSR states [84] (Fig. 7d). For PbPc, the different image potentials experienced by the two conformers, arising from the positively charged metal ion residing at different heights above the substrate, were found to affect both the LUMO energy and the YSR states [91].

In addition, the pronounced sharpness and intensity of YSR resonances provide an order-of-magnitude improvement in both sensitivity and spectral resolution for STM-based vibrational spectroscopy. In the case of PbPc, over 45 vibrational modes were observed, and mode shifts as small as 0.1 meV could be resolved because the technique is not limited by thermal broadening (Fig. 7b) [82].

### Coupled spins in dimers, chains and networks

**Figure 8.**
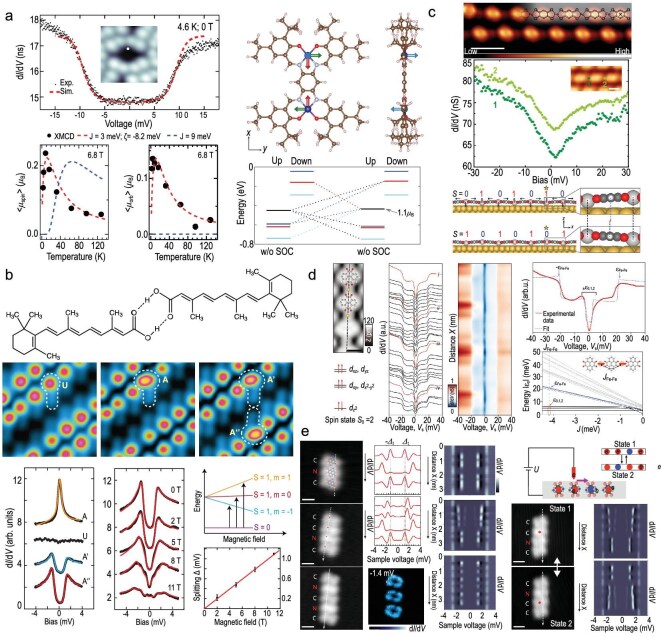
Molecular spin architectures and their tunable interactions. (a) Di-Co complexes spin magnetic excitations in $\mathrm{d}I/\mathrm{d}V$ spectra, temperature-dependent spin and orbital moments (XMCD), and DFT-predicted spin configurations with orbital contributions. Adapted with permission from Li *et al.* [41]. (b) Hydrogen-bonded ReA dimers form nonmagnetic (U), spin-carrying (A) and spin-coupled (A’, A”) states, with STM and $\mathrm{d}I/\mathrm{d}V$ spectra revealing magnetic-field-dependent spin transitions and Zeeman splitting ($g \approx 1.7$). Scale bars: 1 nm and 0.5 nm (inset). Adapted with permission from He *et al.* [43]. (c) STM and models of Fe and Ni chains on Au(111) show ferroelastic Fe chains with $\mathrm{d}I/\mathrm{d}V$ spectra on neighboring atoms, and collective spin-state switching in Ni chains. Adapted with permission from Liu *et al.* [85]. (d) Fe–PTO chain on Ag(111). STM and $\mathrm{d}I/\mathrm{d}V$ spectra at Fe sites show spin excitations. DFT calculations and simulations reveal an $S=2$ ground state and ferromagnetic coupling, with $\pm \varepsilon _{0,1,2}$ dips and $\pm 20$ meV steps. Adapted with permission from Liu *et al.* [86]. (e) TBTAP chains on Pb(111) show end-localized YSR states; STM tip-induced charge transfer enables reversible switching of tetramer configurations. Spatially resolved $\mathrm{d}I/\mathrm{d}V$ maps reveal localization of YSR peaks and redistribution of charge along the chain. Scale bars: 1 nm. Adapted with permission from Li *et al.* [42].

In coupled spin systems comprising two or more magnetic centers, multiple exchange pathways can mediate interspin coupling, including direct covalent exchange, superexchange via hydrogen bonds or bridging ligands, and substrate-mediated indirect interactions. On metallic substrates, the latter may acquire Ruderman–Kittel–Kasuya–Yosida-like character, whereas they can be strongly modified or suppressed on gapped or superconducting surfaces. Advances in on-surface synthesis now enable the atomic-scale construction of dimers, one-dimensional chains and extended networks, while STM/STS provides *in situ* control and spectroscopic access to spin–spin couplings, excitation spectra and collective switching. Figure 8 highlights realizations of these coupling mechanisms.

#### Dimers and orbital anisotropy

Coupled dimers provide a minimal unit for probing exchange interactions. For Co dimers on Au(111), STS revealed spin excitations accompanied by sizeable orbital moments, confirmed by X-ray magnetic circular dichroism and DFT (Fig. 8a) [41]. This highlights the significance of orbital contributions, even in systems considered to be spin-only.

#### Hydrogen-bond-mediated coupling

Non-covalent pathways can also mediate exchange. He *et al.* [43] studied hydrogen-bonded retinoic acid (ReA) dimers on Au(111), identifying nonmagnetic (U), spin-carrying (A) and spin-coupled (A′, A″) states (Fig. 8b). The $\mathrm{d}I/\mathrm{d}V$ spectra revealed Zeeman splitting with $g \approx 1.7$, demonstrating that hydrogen bonds can act as spin conduits.

#### One-dimensional metallo-supramolecular chains

Extending to linear assemblies, Fe and Ni chains on Au(111) display cooperative phenomena. Liu *et al.* [85] observed antiferroelastic ordering in Ni chains, in which sites alternated between $S=1$ and $S=0$ states; tip-induced excitation triggered domino-like collective switching (Fig. 8c). These results emphasize the interplay between elastic strain and spin coupling in low dimensions.

#### Substrate-dependent exchange

Substrate choice can qualitatively alter effective interactions. In Fe–PTO (pyrene-4,5,9,10-tetraone) chains, strong ferromagnetic coupling ($J \approx -4.1$ meV) and high-energy steps (${\sim } \pm 20$ meV) were evident on Ag(111), whereas the same chains on superconducting Pb(111) exhibited strongly suppressed interactions (Fig. 8d) [86]. This contrast reflects the influence of substrate electronic structure on long-range spin correlations.

#### Radical chains on superconductors

Chains of 4,5,9,10-tetrabromo-1,3,6,8-tetraazapyrene (TBTAP) radicals on Pb(111) offer a platform to investigate YSR physics in extended assemblies. STS revealed YSR states localized at the chain ends, while tetramers exhibited bistable charge configurations that could be controlled by tip-induced charge transfer (Fig. 8e) [42]. These experiments demonstrate substrate-mediated coupling and highlight molecular chains as promising binary elements for nanoscale information storage.

In summary, coupled spin systems—ranging from dimers and hydrogen-bonded units to extended chains—illustrate the diversity of exchange pathways and collective responses achievable in molecular assemblies. With advances in on-surface synthesis and spectroscopic resolution, these artificial spin networks provide powerful model platforms for investigating correlated magnetism and for engineering functional quantum architectures.

### On-surface synthesis of radical and open-shell molecules

**Figure 9.**
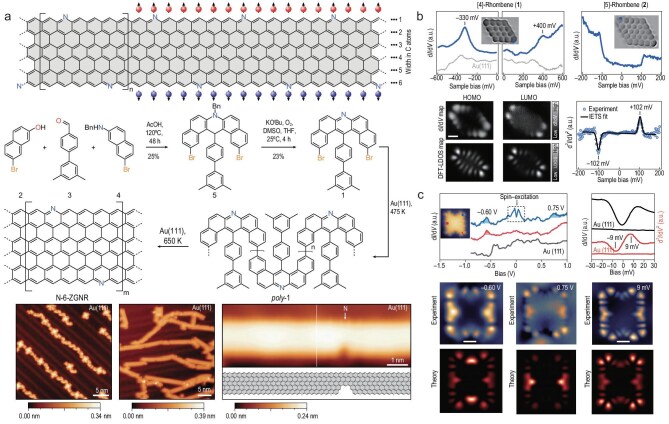
Radical and open-shell molecules realized by on-surface synthesis. (a) N-6-ZGNRs grown from precursor 1; STM shows deposited precursor, cyclized ribbons and a point defect with the nitrogen atom highlighted. Adapted with permission from Blackwell *et al.* [92]. (b) [4]- and [5]-rhombenes; CO-STM and $\mathrm{d}I/\mathrm{d}V$ maps reveal HOMO and LUMO states, with [5]-rhombene exhibiting inelastic spin excitations fitted by a Heisenberg dimer model (10.2 meV). Scale bar: 0.5 nm. Adapted with permission from Mishra *et al.* [17]. (c) ‘Butterfly’ molecule 1 on Au(111); STM/BRSTM, $\mathrm{d}I/\mathrm{d}V$ and IETS spectra reveal electronic states and spin excitations, in agreement with CASSCF singlet–triplet simulations. Scale bars: 0.5 nm. Adapted with permission from Song *et al.* [93].

**Figure 10.**
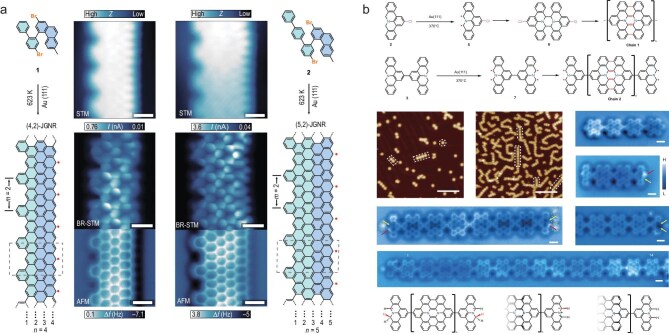
Graphene nanoribbons and diaza-HBC radical chains. (a) (4,2)- and (5,2)-JGNRs; STM, STM with CO tip and nc-AFM resolve the atomic structures and confirm on-surface synthesis. Scale bars: 0.5 nm. Adapted with permission from Song *et al.* [94]. (b) Diaza-HBC chains 1 and 2 on Au(111); STM shows chains of varying lengths, while nc-AFM resolves the chemical structures and terminal units, including methylene radical, azomethine ylide and diradical types. Scale bars: 10 nm (STM); 0.5 nm (nc-AFM). Adapted with permission from Fu *et al.* [95].

Open-shell molecular systems, stabilized by unpaired electrons in $\pi$-radical orbitals, provide highly tunable spin states for quantum magnetism and spintronics. Their delocalized yet chemically controllable nature makes them attractive building blocks for qubits and single-spin devices. On metal surfaces, however, strong hybridization often quenches these states. On-surface synthesis offers a way forward, enabling atomically precise construction of radical-based nanostructures, improved thermal stability and direct access to correlated low-dimensional spin models (Figs 9 and 10).

#### Edge-localized radicals in graphene nanoribbons

Blackwell *et al.* [92] synthesized nitrogen-doped zigzag graphene nanoribbons (N-6-ZGNRs) and observed giant spin splitting at the edges (Fig. 9a). STM and $\mathrm{d}I/\mathrm{d}V$ spectroscopy mapped spin-polarized states confined to the ribbon edges, confirming the robustness of edge-localized radicals.

#### Strong exchange in rhombus-shaped nanographenes

Mishra *et al.* [17] studied zigzag terminated rhombenes, revealing large $\pi$-radical exchange couplings exceeding 100 meV (Fig. 9b). Tunneling spectra exhibited spin excitations consistent with a Heisenberg dimer model, showcasing how edge engineering can maximize magnetic coupling.

#### Frustrated polyradicals

Moving beyond single-edge radicals, Song *et al.* [93] synthesized a butterfly-shaped nanographene with four unpaired electrons (Fig. 9c). Spin-excitation spectroscopy revealed few-meV-scale excitations, and multireference complete active space self-consistent field (CASSCF) calculations confirmed a strongly correlated open-shell singlet ground state, reflecting frustration among multiple radicals.

#### Asymmetric Janus nanoribbons

Further control arises from structural asymmetry. Song *et al.* [94] realized Janus-type GNRs with zigzag edges on only one side (Fig. 10a). Radical states were confined to a single edge, yielding signatures of ferromagnetic alignment and pointing toward directional spin control in ribbon-based devices.

#### Heisenberg spin chains from diaza-nanographenes

Fu *et al.* [95] assembled diaza-hexa-peri-hexabenzocoronene (diaza-HBC) monomers into finite chains that realize antiferromagnetic Heisenberg models (Fig. 10b). Even-length chains displayed finite spin gaps with singlet ground states, whereas odd-length chains exhibited Kondo resonances characteristic of unpaired $S=1/2$ edge spins. Extracted exchange couplings (*J* in tens of meV) match theoretical expectations for ideal spin-1/2 chains. Radical topology and synthetic precision are fundamental design parameters governing correlated behavior in open-shell $\pi$ systems. With continued advances in on-surface synthesis and high-resolution spectroscopy, radical nanographenes and related open-shell molecules are emerging as programmable test beds for low-dimensional magnetism and correlated quantum phases at the atomic scale.

## CHALLENGES AND LIMITATIONS

Despite significant advances in single-molecule and low-dimensional magnetic systems, substantial challenges remain in translating these discoveries into broadly applicable principles and functional devices.

### Intrinsic variability in molecule–surface interactions

As discussed earlier, molecular spin states are exquisitely sensitive to local hybridization, ligand-field distortions and charge transfer. Experimental examples—from Co-porphyrins on Au(111) [22], to zigzag graphene nanoribbons [92], and radical chains on superconductors [42]—demonstrate that even subtle variations in adsorption registry, tip condition or local electrostatics can strongly modify or quench a pronounced Kondo resonance. Such variability poses reproducibility challenges that require thorough statistical mapping, systematic tip calibration and meticulous substrate preparation.

### Low temperatures and ultra-high vacuum conditions

To date, nearly all scanning-probe investigations of molecular magnetism require cryogenic temperatures and ultra-high vacuum (UHV). These conditions suppress thermal decoherence and stabilize fragile spin states but restrict functionality to laboratory environments. At elevated temperatures or pressures, molecular desorption and rapid spin relaxation become dominant. Bridging this gap will require strategies such as designing thermally robust ligands, employing thin decoupling layers or encapsulating spins within protective environments, thereby extending stability into technologically relevant regimes. Encouragingly, proof-of-concept studies beyond scanning-probe microscopy are paving the way: refined chemical design of high-anisotropy lanthanide complexes has pushed magnetic blocking temperatures into the high tens of kelvin, while encapsulation in inert matrices or beneath overlayers has been shown to suppress desorption and improve preservation of spin states outside UHV conditions [96,97].

### Complexity of theoretical modeling

Describing strongly correlated spin phenomena goes well beyond simple Anderson or Heisenberg models. While DFT provides valuable insights into molecular orbitals and adsorption geometries, accurate treatment of Kondo resonances, superconductivity, YSR systems and collective excitations in molecular chains often requires embedding DFT within many-body approaches such as numerical renormalization group or dynamical mean-field theory. For example, combined DFT+NRG calculations have quantitatively reproduced YSR subgap spectra of magnetic molecules on superconducting substrates and their evolution with coupling strength, in direct agreement with STM/STS measurements [98]. Likewise, density-matrix renormalization group calculations have accurately captured finite-size spin gaps and excitation spectra in atomically assembled spin chains, directly reproducing the spectroscopic signatures observed by STM/IETS [99]. At present, no single method offers a unified and predictive description across all regimes, highlighting the need for multi-scale frameworks that can be benchmarked directly against high-resolution spectroscopy data.

### Reproducibility and stability in device-relevant environments

Beyond the fundamental constraints of UHV and cryogenic operation discussed above, molecular spin systems face additional challenges when translated into device contexts. Key issues include ensuring consistent magnetic behavior and readout fidelity across large-area molecular arrays, preserving spin coherence at elevated temperatures, and achieving integration with complementary metal-oxide-semiconductor (CMOS)-compatible microelectronics or two-dimensional materials. Addressing these demands requires targeted solutions—for example, robust anchoring chemistries and protective overlayers for ambient stability, interface-engineering and passivation strategies, and scalable, CMOS-compatible fabrication workflows—which together will be essential to move molecular spintronics toward practical, device-relevant applications.

## FUTURE PERSPECTIVES

Progress at the interface of chemical design, surface engineering and scanning-probe spectroscopy has brought molecular magnetism on surfaces to the verge of device-level functionality. Moving beyond proof-of-principle demonstrations will rely on four converging directions: integration with van der Waals platforms, time-domain control of single spins, operation under technologically relevant conditions and data-driven discovery.

### Data-driven analysis and closed-loop workflows

As spectral features become more complex (spin excitations, Kondo/YSR lineshapes, vibronic progressions), manual fitting becomes a bottleneck. A route forward is physics-informed automation: constrained fits (e.g. Frota/Fano line shapes for Kondo resonances; anisotropy Hamiltonians for inelastic excitations) combined with uncertainty estimates, together with standardized data formats (raw *I*–*V*, lock-in amplitude and phase, and full metadata) to enable cross-laboratory reuse. A critical enabling step will be the widespread adoption of machine-readable, shareable data and metadata to enable robust model benchmarking. Even without introducing new algorithms, such practices will accelerate comparisons across systems and conditions and help disentangle intrinsic molecular variability from instrument- or tip-induced artifacts.

### Superconducting and topological molecular hybrids

Superconducting hosts endow molecular spins with YSR resonances that can be tuned by adsorption geometry, charge environment and intermolecular coupling. Extending from isolated molecules to chains or networks creates band-like YSR spectra and, with appropriate symmetry and coupling, pathways toward topological phases. Superconducting tips—already powerful for Andreev and Josephson spectroscopies—provide phase-sensitive readout channels [42]. A concrete goal is to template well-registered molecular lattices on weakly interacting surfaces, then use local fields and gating to navigate singlet–doublet transitions and control end-localized subgap modes at will.

### Ultrafast and coherent control with scanning probes

Time-domain techniques transform static spectroscopy into true spin dynamics and control. All-electronic pump–probe STM has already resolved nanosecond relaxation and identified dominant decoherence channels at the atomic scale [26]. Building on these ideas, combining pulsed protocols with molecule-specific anisotropy and tailored environments (e.g. superconducting tips) may enable Rabi control, Ramsey-type measurements and time-resolved interrogation of YSR excitations. Recent work has demonstrated that such coherent control on surfaces can be extended beyond single spins, enabling atomic-scale multi-qubit operation in engineered magnetic-atom arrays [100]. The immediate challenge is co-design: molecular ligands and adsorption geometries should be chosen together with local electromagnetic environments to maximize drive efficiency and readout contrast, extending the manipulation toolbox introduced by STM-based methods [22]. Achieving high-fidelity control will further require that researchers integrate pulsed and ESR-style drive structures for coherent manipulation.

### Molecular spins on 2D platforms

Some 2D materials offer atomically flat, chemically benign supports on which molecular spins can be positioned, decoupled and addressed. Ultrathin insulating spacers (e.g. monolayer insulators) reduce hybridization and spectral broadening while retaining tunneling access, thereby stabilizing spin excitations and Kondo/YSR phenomenology under weaker molecule–substrate coupling. However, the relative inertness of many layered substrates can be a double-edged sword: weak molecule–substrate coupling helps preserve intrinsic spin properties but can also hinder stable adsorption and STM access, even at low temperature. For this reason, ultrathin insulating spacers (e.g. NaCl, h-BN or oxides) remain a widely used and practical route to reduce hybridization while retaining tunneling access and stabilizing spin excitations and Kondo/YSR phenomenology. In addition, on-surface synthesis and site-selective assembly on such buffers provide a practical route to fix molecular geometry and connectivity, thereby improving reproducibility and enabling scalable coupled-spin architectures. Engineered electrostatics (local gates, work-function patterns) and controlled strain still provide useful levers over magnetic anisotropy and exchange, and a realistic mid-term target is the assembly of ordered molecular arrays on insulating buffers with lithography-defined gates, enabling *in operando* tuning of charge and spin states together with robust spectroscopic addressability [48]. To systematically exploit these platforms, key design considerations include using ultrathin insulators to tune hybridization and sharpen spectroscopic fingerprints, and co-engineering local electrostatics and ligand fields to place spin excitations or YSR resonances in accessible bias windows.

### Toward ambient and device-relevant operation

Most single-molecule studies to date rely on cryogenic temperatures and UHV. Bridging to practical conditions will likely require three complementary strategies. First, *intrinsically robust* open-shell frameworks (e.g. nanographenes and radical polymers), whose magnetic couplings remain large when partially hybridized with surfaces. Second, *passivation and encapsulation* via ultrathin insulating layers to suppress desorption and chemical aging while retaining spectroscopic access [48]. Third, *hybrid readout* approaches that combine different scanning-probe modalities under controlled UHV conditions, such as integrating MExFM with tunneling or spin-sensitive spectroscopy, offer complementary access to atomic-scale electrical and magnetic information. In parallel, nanoscale field-sensing techniques developed for ambient environments, including nitrogen-vacancy center magnetometry and miniature Hall probes, represent a promising direction for extending such readout concepts beyond UHV systems in the future. A mid-term milestone is ambient-compatible readout of a subset of molecular arrays assembled and characterized at low temperature, then preserved by capping or transfer.

## CONCLUSIONS

Surface-supported molecular magnetism now offers chemically programmable, atomically precise access to correlated spin phenomena, ranging from Kondo screening and magnetic anisotropy to YSR states. The path ahead emphasizes translation while continuing active fundamental research. Van der Waals interactions for stability and control [48], ultrafast and ESR protocols for coherent manipulation [26], complementary readout and passivation for device relevance, and standardized, data-driven analysis for speed and reproducibility will be key. With these threads aligned to multi-scale modeling, the field is poised to move beyond iconic case studies toward programmable molecular architectures for quantum sensing, simulation and information processing.
